# Design of a silicon Mach–Zehnder modulator via deep learning and evolutionary algorithms

**DOI:** 10.1038/s41598-023-41558-8

**Published:** 2023-09-05

**Authors:** Romulo Aparecido de Paula, Ivan Aldaya, Tiago Sutili, Rafael C. Figueiredo, Julian L. Pita, Yesica R. R. Bustamante

**Affiliations:** 1 Center for Advanced and Sustainable Technologies, State University of Sao Paulo (UNESP), São João da Boa Vista, SP 13876-750 Brazil; 2 Centre for Research and Development in Telecommunications (CPQD), Campinas, SP Brazil; 3 Department of Electrical Engineering, École de Technologie Supérieure (ÉTS), Montreal, QC H3C 1K3 Canada; 4 Department of Electronic and Electrical Engineering, University College London (UCL), Gower St, London, WC1E 6BT UK; 5 Infinera Unipessoal Lda, Carnaxide, Portugal

**Keywords:** Electrical and electronic engineering, Engineering, Optics and photonics, Integrated optics, Optoelectronic devices and components

## Abstract

As an essential block in optical communication systems, silicon (Si) Mach–Zehnder modulators (MZMs) are approaching the limits of possible performance for high-speed applications. However, due to a large number of design parameters and the complex simulation of these devices, achieving high-performance configuration employing conventional optimization methods result in prohibitively long times and use of resources. Here, we propose a design methodology based on artificial neural networks and heuristic optimization that significantly reduces the complexity of the optimization process. First, we implemented a deep neural network model to substitute the 3D electromagnetic simulation of a Si-based MZM, whereas subsequently, this model is used to estimate the figure of merit within the heuristic optimizer, which, in our case, is the differential evolution algorithm. By applying this method to CMOS-compatible MZMs, we find new optimized configurations in terms of electro-optical bandwidth, insertion loss, and half-wave voltage. In particular, we achieve configurations of MZMs with a $40~\text {GHz}$ bandwidth and a driving voltage of $6.25~\text {V}$, or, alternatively, $47.5~\text {GHz}$ with a driving voltage of $8~\text {V}$. Furthermore, the faster simulation allowed optimizing MZM subject to different constraints, which permits us to explore the possible performance boundary of this type of MZMs.

## Introduction

The popularization of multimedia streaming and internet of things (IoT) services, alongside the migration to a distributed computing and storage paradigm, has leveraged the transmission capacity requirements that network operators must satisfy^1^. Meeting such capacity demands is particularly challenging in short-range applications, where networks are subject to stringent cost constraints. This is the case, for instance, of high-speed optical interconnects (OIs) that enable connectivity between geographically distributed hyper-scale data-centers^2,3^. One of the critical elements of this type of system, both from the point of view of cost and performance, is the optical transmitter, on which the electro-optical modulator plays a fundamental role^4^. The well-known lithium niobate (LiNbO$_3$ or LN) modulators, which has been extensively employed in long-haul and metropolitan systems, presents high performance, but cannot be efficiently integrated with the associated electronics, also requiring a large footprint and expensive raw materials^5^.

In this context, integrated photonics has attracted significant attention in recent years. In particular, silicon (Si) photonics has emerged as a high-potential platform for implementing low-cost and high-performance optical modulators, since its compatibility with complementary metal-oxide-semiconductor (CMOS) enables not only the aforementioned monolithic integration with the electronic stage, but also to take advantage of its fabrication know-how and the mature manufacturing infrastructure^6^. However, in contrast to LiNbO$_3$, Si has a centrosymmetric crystalline structure, which leads to weak parametric electro-optic effects (i.e., Pockels and Kerr effects). On the other hand, the semiconductor nature of Si allows injection and extraction of free carriers that can be exploited to build phase shifters based on the plasma dispersion effect (PDE)^7^. This phenomenon allows the electronic control of the structure refractive index, enabling one to implement rib-waveguide-based phase shifters, which are a fundamental constitutive block of in-phase and quadrature optical modulators (IQMs)^8^. Silicon phase shifters can be employed to control the interferometric patterns in different interferometer configurations, such as micro-ring resonators (MRR), Michelson modulators, and Mach–Zehnder modulators (MZM)^9^. Although both MRRs and Michelson interferometer modulators (MIM) exhibit a compact area, low power consumption, and high modulation efficiency, they suffer from a limited modulation bandwidth^10,11^. Alternatively, for high-speed systems, albeit their relatively large footprint and high power consumption, MZMs present the best trade-off between modulation bandwidth, consumption, and insertion loss^12^. Furthermore, MZMs show additional advantages over MRRs and MIM, such as improved thermal tolerance and significant reduction of the chirp imposed to the modulated signal^13^.

The power consumption of the MZM is typically quantified in relation with the voltage required to induce a phase shift of $\pi ~\text {radians}$ between the two interferometric arms, which is often represented as $V_\pi$. The value of $V_\pi$, therefore, depends on whether the MZM has a single phase, only on one interferometric arm, or two phase shifters, one in each arm. However, high performance MZMs usually adopt the second approach as this results in half $V_\pi$ for each phase shifter. Values of $V_\pi$ lower than $1~\text {V}$ have been achieved in double-arm silicon-based MZMs employing PDE with carrier injection^14^. However, the slow carrier injection dynamics limited the performance of the first devices designed based on this principle, presenting a relatively low bandwidth, in the order of hundreds of megahertz^15^. Even if significant progress has been made on carrier-injection MZM, such as the introduction of an resistance and capacitance (RC) equalizer, this configuration is generally outperformed by MZMs based on carrier extraction^16^. Alongside with the carrier dynamics, the bandwidth of carrier-extraction MZMs is limited by the interaction of the junction with the driving electrodes, which impact on the RF losses, the RF and optical waves velocity matching, and the impedance matching between the electrical source, the transmission line and the termination^17^. Aiming to increase the modulation efficiency, traveling-wave electrodes (TWEs) were implemented, thus extending the interaction length between the optical and the electrical signals. In particular, TWEs with ’T’-shaped extensions, namely slow-TWEs, can be used to increase the RF refractive index and improve the velocity matching between the RF and the optical waves^18^. Moreover, series push-pull (SPP) driving configuration can minimize RF losses by reducing the junction capacitance by half and doubling the junction resistance. In addition, a slight impedance mismatch can be implemented between the electrode and the termination to further extend the MZM bandwidth^19^.

In this context, different devices relying on carrier extraction and using slow-TWE have been reported. For instance, in^17^ a device with a $3\text {-dB}$ modulation bandwidth of $41~\text {GHz}$ was reported. Nevertheless, such a large bandwidth was achieved at the cost of a value of $V_\pi$ as high as $11.4~\text {V}$. Alternatively, by optimizing the doping profile and the optical and the RF waveguides design, in^20^ a $6\text {-dB}$ modulation bandwidth of $50~\text {GHz}$ at a $2~\text {V}$ reverse bias and a $V_\pi$ of $6.3~\text {V}$ was demonstrated. Further improvements were presented in^19^, where an impedance mismatch between the traveling wave electrode and the on-chip termination was deliberately introduced, achieving a $3\text {-dB}$ modulation of $46~\text {GHz}$ with a $V_\pi$ of $7.6~\text {V}$ and an insertion losses (IL) of $8.4~\text {dB}$. As can be perceived from the aforementioned works, larger modulation bandwidth is achieved at the expense of higher power consumption and IL. Following an alternative approach, a substrate-removed MZM with a modulation bandwidth exceeding $50~\text {GHz}$ was reported in^21^, but this type of structure hinders the fabrication process and increases the sensitivity of the device to mechanical vibrations. On the other hand, in^22^ the authors explored the adoption of segmented MZMs, in which a distributed driver feeds different segments of phase shifters, thus decreasing the microwave loss and increasing the modulation bandwidth up to $45~\text {GHz}$, while keeping $V_\pi$ below $10~\text {V}$. However, segmented MZMs presents several drawbacks, as its inefficient phase matching between the driving signals and the low modulation gain compared to conventional phase shifters. Alternatively, another approach to enhance the performance of phase shifters is employing a slow-light guiding structure, in which a photonic crystal arrangement is utilized as the optical waveguide^23^. However, the main challenge of this alternative is its sensitivity to fabrication errors since small variations of the features lead to high optical losses. Furthermore, the inclusion of ring-resonators in one or both arms of a Mach-Zehder interferometer has also been proposed to improve modulation efficiency^24^. Nevertheless, this structure inherits the high thermal sensitivity and the bandwidth limitation of ring resonators, limiting its overall performance. Finally, it is important to report recent works exploring the combination of Si with other materials to improve the MZM overall performance. For instance, in^25^ a highly-nonlinear polymer is used to generate the Pockels effect not present in silicon. This organic-silicon hybrid device achieved a bandwidth of $40~\text {GHz}$ with a $V_\pi$ as low as $1.46~\text {V}$ and an IL of $0.7~\text {dB}$. Unfortunately, the use of a nonlinear polymer is not CMOS-compatible, making its manufacturing more complex and increasing its cost. Alternatively, graphene has also been proposed to implement phase shifters. However, even if low $V_\pi$ can be achieved, simultaneously attaining a low driving voltage and broad modulation bandwidth is still difficult^26^. In addition, the integration of 2D-material, such as graphene, requires careful manipulation and the integration with the silicon layer is still an unresolved barrier. Therefore, due to its natural compatibility with CMOS manufacturing process and general performance, standard TW-MZM configuration continues being the focus of different works.

Since the performance of a TW-MZM highly depends on its constitutive integrated-phase shifters, its optimization becomes critical to achieve a competitive performance. Nevertheless, the large number of design parameters (including section dimensions, doping concentrations, and bias voltage), alongside the complex simulation (accounting for both electrical and optical fields and their interrelation) makes the optimization process challenging and computationally expensive. Consequently, brute force optimization is generally unfeasible, requiring extremely powerful and expensive computational platforms. Even state-of-the-art heuristic optimization algorithms require an elevated number of iterations, leading to large processing times. Since the most computational expensive stage of these optimization algorithms is the electromagnetic simulation of the solution candidates, which requires the 3D simulation of the optical structure, we propose to substitute it by a lower complexity artificial neural network (ANN)-based model, significantly reducing the time required to optimize this structure. For that, in this work we develop an accurate ANN-based model of an integrated carrier-extraction TW-MZM and use it in combination with a well-established heuristic optimization method, i.e. the differential evolution (DE), to find different high-performance configurations, allowing us to design this devices with better performance with lower computational cost. The remaining of this paper is organized as follows: “ANN-basedmodel of the MZM” section introduces the device to be optimized and describes the develop ANN-based model, including its architecture, training, and prediction performance; “Optimization of the MZM employing differential evolution” section briefly present the DE algorithm and apply it in combination to the developed ANN-based model to obtain different configurations; finally, in “Conclusions” section, the most relevant conclusions are drawn.

## ANN-based model of the MZM

In the present section we describe the modeling of an integrated TW-MZM using ANNs. In “Device to be optimized” section, we introduce the device to be optimized, including the design parameters, and the chosen figures of metrics. Following, the dataset obtained using electromagnetic simulations is described in “Simulation of random configurations” section. Then, the adopted ANN architecture and its particularities are presented in “ANN model and training” section. Finally, in “Analysis of the model prediction accuracy” section, the prediction accuracy of the developed model is quantified and discussed.

**Table 1:** Optimization variable and design parameters.

Optimizable parameters			Fixed parameters		
Parameter	Range	Unit	Parameter	Value	Unit
$V_{bias}$	$-10$ up to $-2.5$	V	p concentration	$3\times 10^{17}$	cm$^{-3}$
$W_C$	450 up to 500	nm	n concentration	$3\times 10^{17}$	cm$^{-3}$
L	0.5 up to 4	mm	p+ concentration	$4\times 10^{18}$	cm$^{-3}$
$W_{p-slab}$	50 up to 500	nm	n+ concentration	$4\times 10^{18}$	cm$^{-3}$
$W_{pp-slab}$	600 up to 1000	nm	p++ concentration	$1\times 10^{20}$	cm$^{-3}$
$W_{n-slab}$	50 up to 500	nm	n++ concentration	$1\times 10^{20}$	cm$^{-3}$
$W_{nn-slab}$	600 up to 1000	nm	Waveguide height	220	nm
$PN_{offset}$	$-225$ up to 225	nm	Slab height	61	nm

### Device to be optimized

**Figure 1:**
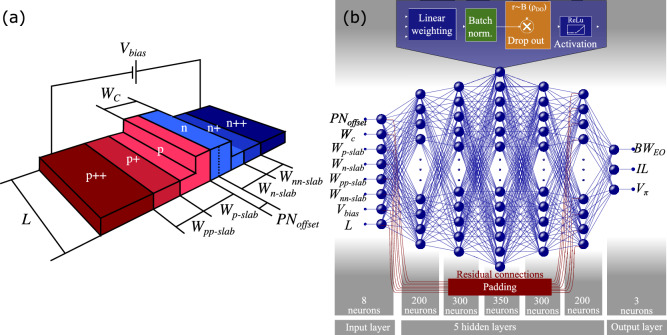
(**a**) 3D view of the PM to be optimized indicating the different optimization variables. (**b**) Five-layer MLP model designed to emulate the electromagnetic simulation model, with the indication of the number of neurons per layer. A skip connection is set between the input layer and the last hidden layer, demanding a padding to equate the array’s size. The figure also shows the neuron operation, on which the linear transformation is the first block, followed by the BN, the DO and, finally, the nonlinear activation ReLu function.

The device to be optimized is an integrated MZM employing PDE-based phase shifters. In particular, we considered a modulator operating in carrier extraction mode equipped with TWEs, as this configuration presents the best overall performance for large-bandwidth MZMs. More specifically, since the performance of such modulator is fundamentally determined by the properties of the phase shifters in each arm, the optimization process will be focused on these elements. Figure 1a shows the PIN rib waveguide used to implement the phase shifter, with the structure biased at $V_{\text {bias}}$ and being defined by a length *L*, a waveguide width $W_C$, and presenting six different regions with different doping concentrations and widths. In specific, this regions are defined by the variables $W_{\text {pp-slab}}$, $W_{\text {p-slab}}$, $W_{\text {n-slab}}$, $W_{\text {nn-slab}}$ that corresponds to the widths of the p+, p, n, and n+ regions, respectively, and the offset of the PN interface, $PN_{\text {offset}}$. Moreover, the doping concentration of each region, as well as the waveguide and slab heights, are not subject to optimization since they are usually fabrication constraints imposed by the foundry. In Table 1, the ranges of the optimization variables and other design parameters are summarized.

Different performance metrics can be used to assess the performance of a MZM, among them we can highlight three widely adopted metrics that usually are employed in a complementary manner. The first metric is the optical insertion loss (*IL*), which depends on the length of the structure and the amount of carriers within the modal area, in consequence also depending on the bias voltage. Next, the electro-optical bandwidth ($BW_{\text {EO}}$) is also an important assessment parameter, defining the modulator baud rate limits in high-capacity optical systems. Finally, the voltage required to impose a phase shift of $\pi$ radians is critical since it determines the device power consumption, which is becoming an increasing concern. However, the importance of each metric should be weighted based on the particular application requirements and limitations.

### Simulation of random configurations

**Figure 2:**
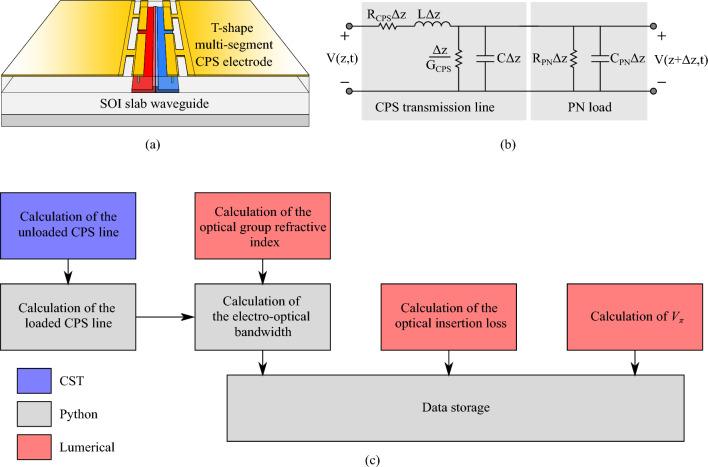
(**a**) Representation of the simulated phase shifter, (**b**) equivalent transmission-line model and (**c**) block diagram of the employed model to assess the $BW_{\text {EO}}$, *IL*, and $V_\pi$. Blue indicates the part of the model implemented in CST whereas gray and red blocks represent implementation in Python and Lumerical, respectively.

**Figure 3:**
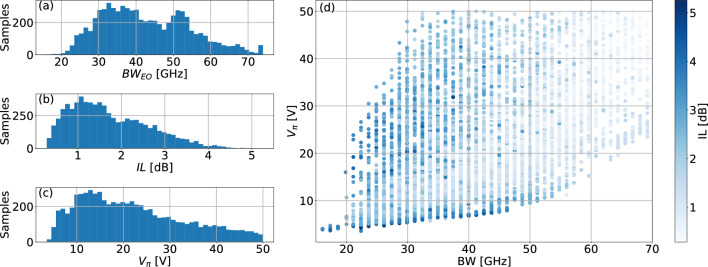
Modulators dataset utilized to train and test the ANNs: (**a**), (**b**) and (**c**) illustrates the histograms of the dataset in relation to $BW_{\text {EO}}$, *IL*, and $V_\pi$; (**d**) shows $V_\pi$ in relation to the $BW_{\text {EO}}$, with the *IL* given in a blue color scale.

The first stage to develop an ANN-based model is to build a dataset composed of randomly generated representative MZM configurations. For that, each configuration corresponds to a combination of the optimization parameters randomly chosen within the limits listed in Table 1. To assess the MZM performance under these conditions, each of these configurations was simulated by combining specific commercial software packages, i.e. CST Microwave Studio^27^ and Lumerical^28^, and high-level programming language (Python). Figure 2a shows the schematic of the simulated phase shifter equipped with T-shape multi-stage slow-wave traveling wave electrodes implemented on the coplanar stripline (CPS) technology. In Fig. 2b, we show the equivalent transmission line model of the phase shifter, which includes both the CPS electrode and the PN load. In order to integrate this model, first of all, the parameters of the unloaded CPS line were calculated using CST Microwave Studio, which is capable of calculating both the transmission line impedance and the RF losses. These parameters were then used to calculate using a high-level programming language the parameters of the loaded CPS line, where the intermediate PN section is considered employing the model proposed in^8^. The parameters of the loaded CPS line, alongside the optical group refractive index calculated using Lumerical Mode, were used to calculate the electro-optical bandwidth $BW_{\text {EO}}$. Regarding the computation of both $V_\pi$ and the *IL*, the Lumerical Device module was used. The detailed semi-analytical model is described in^29^. The block diagram of the co-simulation environment is shown in Fig. 2c. Since the two arms of the MZM are equal, the design process is reduced to the phase shifter of each arm. In total, the datasets are composed of 10,000 MZM configurations, which histograms obtained for the $BW_{\text {EO}}$, *IL*, and $V_\pi$ are shown in Fig. 3a–c, respectively. As can be seen, most of the random configurations present *IL* values close to $1.25~\text {dB}$, with $V_\pi$ distributed around $12.5~\text {V}$. Furthermore, the $BW_{\text {EO}}$ histogram is characterized by two well-defined peaks, one centered at approximately $30~\text {GHz}$, with a second narrower peak at $50~\text {GHz}$. Thus, most of the samples present IL, $V_\pi$, and $BW_{\text {EO}}$ values concentrated in an average region, where none of the individual metrics is excessively penalized. In order to achieve more uniform histograms, the design parameters can be engineered instead of considering uniform randomly generated values. Although these histograms give valuable information about the aforementioned metrics, they do not give any knowledge on the relation between them. Thus, to understand this relation, in Fig. 3d, we show the values of $V_\pi$ in terms of the $BW_{\text {EO}}$ with the *IL* as a color scale for each MZM configuration present on the here employed dataset. The first point to note is a lower bound in the $V_{\pi }$ versus $BW_{\text {EO}}$ relation, indicating the expected trade-off between these parameters. Moreover, in this boundary it is possible to notice high *IL* values, not being the most viable solution for the proposed MZM design and justifying the need for the proposed optimization methodology. Finally, the dataset was split into training and test subsets, being the former composed of 9,000 samples, whereas the remaining 1,000 samples compose the latter.

### ANN model and training

To model the MZM, the employed ANN architecture was a fully-connected multi-layer perceptron (MLP), which is widely adopted due to its versatility and efficient training^30^. The MLP had eight inputs and three outputs corresponding to the design variables and the three figures of merit. Moreover, ANNs with different amounts of hidden layers were tested, in particular with 4, 5, and 6 layers, here denoted as MLP$_4$, MLP$_5$, and MLP$_6$, respectively. The number of neurons of each layer, alongside with the number of model hyperparameters and the total neuron count, are listed in Table 2. Furthermore, we adopted the rectified linear unit (ReLU) as activation function in all neurons and the root mean squared (RMS) error as cost function between the output of the ANN and the desired values for the optimization metrics. Next, regarding the ANN training, the Kaiming uniform method was used to initialize the synaptic weights^31^ and the decoupled weight decay Adam optimizer (AdamW) with $\beta _1~=~0.9$ and $\beta _2~=~0.999$ was employed to optimize the cost function. Additionally, two versions of each MLP configuration were implemented. The base version of the MLPs considers no additional features. The full version of the MLPs, on the other hand, considers the following three improvements: Connection dropout (DO)^32^, Batch normalization (BN)^33^ and Residual connections (RCs)^34^.

**Table 2:** Summary of the proposed ANNs, including its architecture characteristics, activation function, and training parameters.

		Parameters	Parameters with BN	Neurons	1st Layer	2nd Layer	3rd Layer	4th Layer	5th Layer	6th Layer
Architecture	MLP$_4$	213203	215203	1000	200	300	300	200	—	—
	MLP$_5$	333553	336253	1350	200	300	350	300	200	
	MLP$_6$	456403	459803	1700	200	300	350	350	300	200
Act. function	ReLu									
Cost function	MSE									
Training	Initialization					Kaiming uniform method				
	Optimizer					AdamW				
						W$_d$ parameter			0.05	
						$\beta _1$ parameter			0.9	
						$\beta _1$ parameter			0.99	
Improvements	Dropout					$\checkmark$				
	Batch normalization					$\checkmark$				
	Residual connections					$\checkmark$				

The implemented improvements are also included.

**Figure 4:**
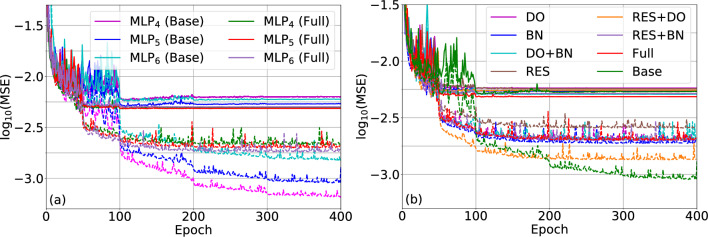
MSE loss, in logarithmic scale, of the training set (dashed lines) and the validation set (straight lines) during the DNN training for: (**a**) the three investigated ANNs architectures (i.e., MLP$_4$, MLP$_5$, and MLP$_6$) in its ‘base’ and ‘full’ configurations (i.e., without and with DO, BN, and RC, respectively); and, (**b**) the MLP$_5$ configuration with all the possible combinations of DO, BN, and RC.

In addition to ANNs with different numbers of hidden layers (denoted as MLP$_4$, MLP$_5$, and MLP$_6$), we also considered variations in which the improvements DO, BN, and RCs are implemented or not. In particular, we denote as ‘base configuration’ the ANN in which none of the aforementioned improvements is implemented. The ‘full configuration’, on the other hand, stands for the configuration considering simultaneously DO, BN, and RCs implementation. In Fig. 1b, we show one of the considered ANN configurations, i.e. full MLP$_5$, where, for illustration purposes, the DO and BN blocks and the RCs are highlighted in different colors.

**Figure 5:**
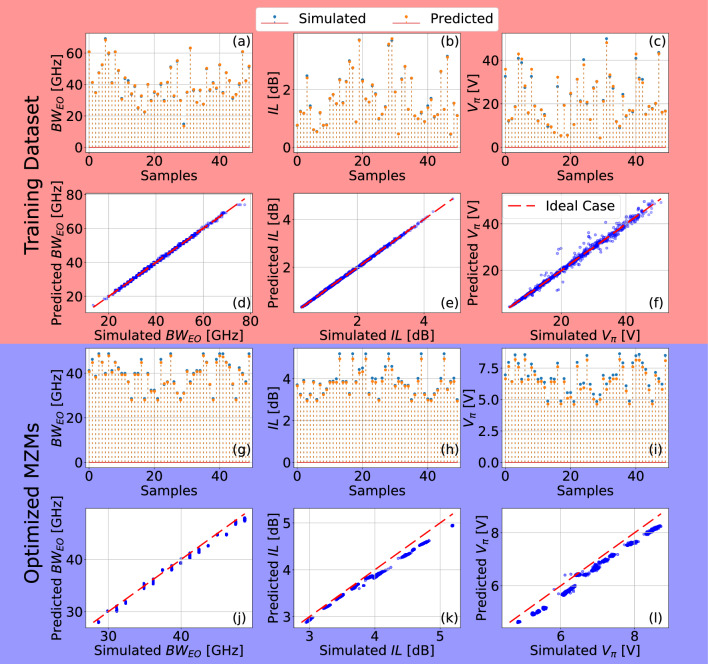
Accuracy results of the DNN modeling. (**a**)–(**f**) show the comparative of the ANN modeling in relation to the test dataset. (**a**), (**b**), and (**c**) show a comparative between the DNN prediction and the simulation setup results, for the $BW_{\text {EO}}$, IL and $V_\pi$, respectively. We utilized 50 random samples for this comparative. (**d**), (**e**) , and (**f**) show the relation between simulated and predicted performance parameters, accounting the entire test set. It also displays the comparison of the values of the metrics of the optimized MZMs obtained by the ANN-based model and by electromagnetic simulation. (**g**)–(**l**) show the comparative of the ANN modeling and the simulation model results for the optimisation method obtained MZMs. (**g**), (**h**) and (**f**) present the $BW_{\text {EO}}$, *IL*, and $V_\pi$ of 50 samples, respectively. Whereas in (**j**), (**k**) and (**l**) it is shown the values of $BW_{\text {EO}}$, *IL*, and $V_\pi$ obtained by the ANN model in terms of the simulated values for the whole set of 1,700 optimized configurations. The ideal relation is plotted as a dashed line, and each point is a MZM.

In order to assess the training and the effect of the combination of DO, BN, and RCs on it, in Fig. 4, we show the MSE curves in terms of the iteration number (epoch) for both the training (dashed lines) and validation (straight lines) sets, considering different ANN configurations. In particular, in Fig. 4a, we present the loss functions for the base and full configurations of MLP$_4$, MLP$_5$, and MLP$_6$. As can be seen, in the initial stage, the MSE curves of the training and validation follow a clear downward tendency. However, as expected, as the number of epoch increases, the values of the training and validation MSE saturate. Moreover, comparing the training and validation MSE curves, independently of the ANN configuration, the loss function of the training subset remains decreasing for a larger number of epochs, tending to lower values than for the validation subset. Nevertheless, when we compare the training and validation MSE curves in terms of the ANN configuration, as shown in Fig. 4b, it is possible to observe that some configurations lead to higher training MSE but lower validation MSE, which indicates that the model is less affected by overfitting. Thus, among the investigated configurations, the full MLP$_5$ is the one showing the lowest impact of the overfitting, presenting training and validation MSEs after 400 epochs of $2.07 \times 10^{-3}$ and $4.85\times 10^{-3}$. Furthermore, to isolate the effect of DO, BN, and RCs in the training performance, Fig. 4b presents the ‘base’ and ‘full’ configurations for the MLP$_5$ compared to implementations considering all the possible combinations of the ANN improvements here considered. Comparing the configurations implementing just DO, BN, or RCs, the first improvement shows the higher performance gains in terms of validation MSE. As can be seen, this configuration also presents a high training MSE, which indicates that, as expected, DO indeed tackles partially the effect of overfitting. When two of the three enhancement techniques are applied, we found that the combination of DO and BN leads to the best performance. This performance is further enhanced when we implement simultaneously DO, BN, and RCs, resulting in the ‘full’ configuration here proposed. In summary, this analysis reveals that an intermediate number of hidden layers (i.e., five characterizing the MLP$_5$ architecture), and the simultaneous adoption of DO, BN, and RCs lead to the optimum validation performance among all considered configurations. Thus, this ANN configuration will be employed on the MZM optimization process discussed in “Optimization of the MZM employing differential evolution” section.

At last, it is important to highlight that, while the electromagnetic simulation model (i.e., the here employed Lumerical Device and Model modules) takes about 3 minutes to evaluate a MZM structure on an Intel Xeon E5-2650 CPU, the ANN takes only $17~\mu \text {s}$ to predict the proposed metrics parameters. It is important to highlight that these times were calculated performing 1000 evaluations and dividing the total required time by the number of runs. Considering the obtained computational complexity reduction, we can now execute an heuristic algorithm, such as DE, taking into consideration large size populations and high number of iterations for the MZM optimization, increasing the possibility to achieve the optimum configuration for a given set of requirements.

**Figure 6:**
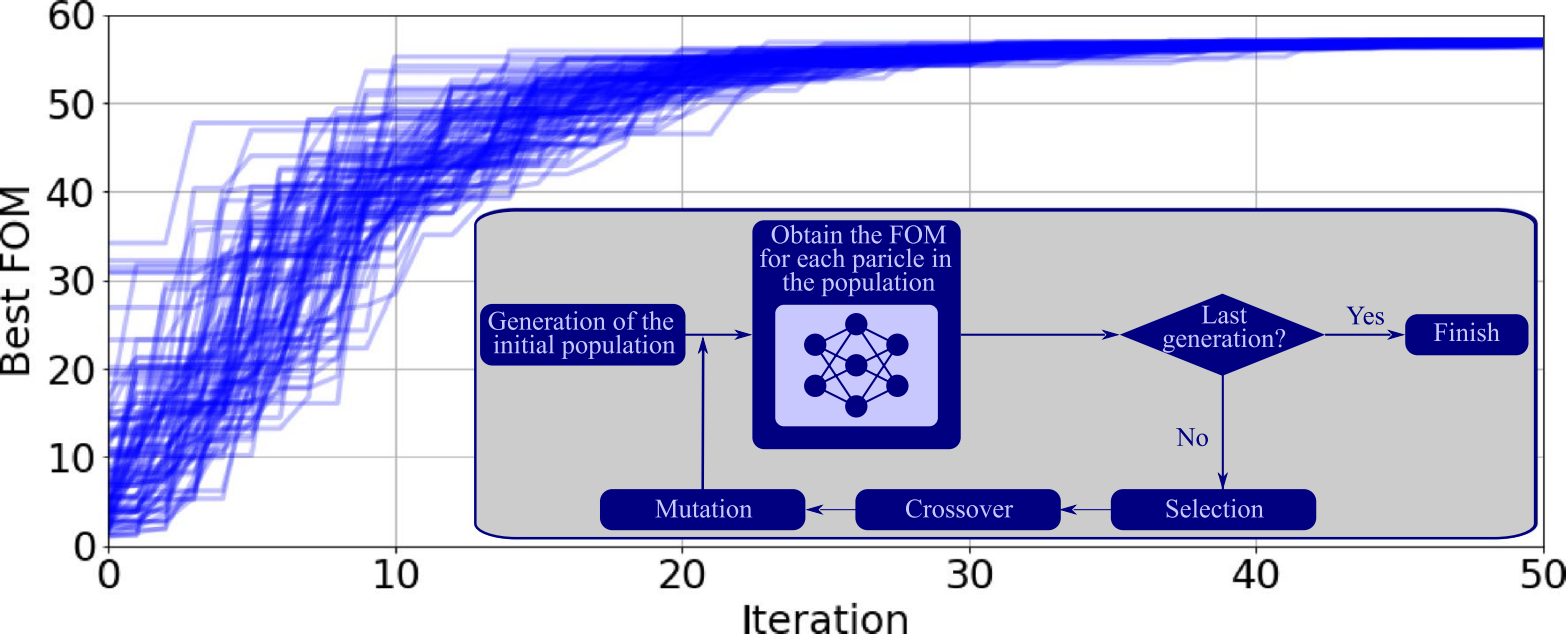
Figure of merit (FOM) of the best individual in each generation for each iteration taking into account 100 different initialization configurations. In addition, as an inset, the flowchart of the DE optimization algorithm, in which the FOM (fitness) is estimated via the ANN-based model for the MZM.

### Analysis of the model prediction accuracy

The MSE adopted here as the analysis cost function considered the average value of the errors of the three MZM considered performance metrics (i.e., $BW_{\text {EO}}$, *IL*, and $V_\pi$). Therefore, the average MSE cannot be used to assess the accuracy of the trained ANN to predict the isolated ANN accuracy for each metric. In order to analyze the capability of the model to predict each of these metrics, in Fig. 5a–c, we present a set of 50 samples of randomly selected MZM configurations from the validation subset to compare the outcomes of the proposed ANN model to the output of the Finite-Difference Time-Domain (FDTD) simulation. Thus, Fig. 5a–c, reveal that, as expected, the outputs of the ANN are qualitatively very close to the simulated values for the three considered MZM performance metrics. To ensure that the improved performance is not an artifact introduced by the ANN and that it really corresponds to optimized MZM configurations, the found configurations were simulated using the commercial FDTD simulator. Therefore, in Fig. 5d–f, we show the output of the ANN-based model in comparison with the values obtained through the electromagnetic simulation model in terms of $BW_{\text {EO}}$, *IL*, and $V_\pi$, respectively. As can be observed, each metric is accurately predicted by the developed model, highlighting the $BW_{\text {EO}}$ and *IL* predictions, which meet almost perfectly the simulated values. However, in comparison, the $V_\pi$ ANN-based predictions present a higher error, especially for $V_\pi$ values above 25 V, which are not usually desired in MZM designs as they are extremely high. Overall, the MSE values obtained for the $BW_{\text {EO}}$, *IL*, and $V_\pi$ performance metrics are 0.23, $5\times 10^{-4}$ and 1.56, respectively, and the Pearson correlation coefficient is above $98\%$ for all metrics.

## Optimization of the MZM employing differential evolution

In this section, we describe the employment of the proposed ANN-based MZM model jointly with the DE optimization algorithm to design optical modulators considering the proposed performance metrics. For that, initially, the optimization methodology is described in “DE optimization employing ANN-based model” section, detailing the DE algorithm and its integration with the ANN-based models. Afterwards, in “Optimization of the MZMs for different performance metrics” section, we show that the proposed approach can effectively find MZM configurations that cannot be obtained by random selection of parameters unless a prohibitively large number of configurations are tested, being a viable solution for the design of integrated optical modulators.

**Figure 7:**
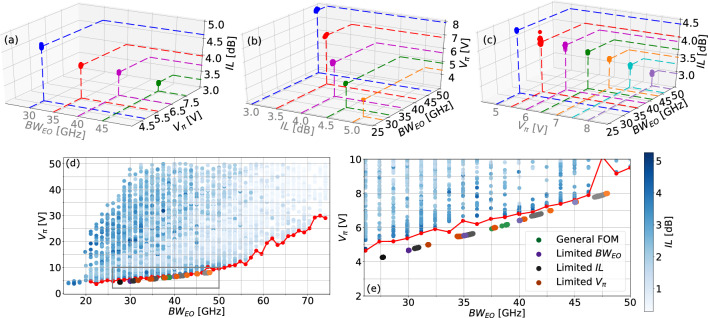
Performance metrics for the 1500 attained MZMs using DE optimization. The optimization was carried out considering the variation of only two metrics, while the (**a**) $BW_{\text {EO}}$, (**b**) *IL*, and (**c**) $V_\pi$ values were limited to those illustrated in the gray axis. Each cluster is composed of 100 optimized MZMs. (**d**) ANN training set and the Pareto front related to the minimum $V_\pi$ and the maximum $BW_{\text {EO}}$. (**e**) Amplified region with MZMs configurations attained via DE optimization, on which different colors indicate different optimization conditions.

### DE optimization employing ANN-based model

Once the ANN model of the integrated MZM has been trained and validated, it can be employed to substitute the more computationally complex electromagnetic simulation model in the figure of merit (FOM) assessment within the desired optimization algorithm^35^. In particular, we chose the differential evolution (DE) algorithm due to its trade-off between optimization and computational complexity^36^, being an iterative heuristic optimization method that emulates the natural evolution of the species. For that, an initial population of *N* tentative solutions (denominated ‘individuals’) undergoes an iterative process in which the best individuals in terms of the cost function are selected, combined (crossover stage), and randomly modified (differential mutation stage) to generate a new population that outperforms the precedent one, as depicted in the flow diagram in the inset of Fig. 6. Particularly, in the present work, we employed a population size of 1000 elements, a single-point crossover with a probability of 0.7, and a mutation intensity of 0.5. Moreover, in order to avoid any dependency on the initialization, we considered 100 independent initial populations.

Aiming to observe the refinement of a given figure of merit through the DE optimization process, in Fig. 6 we show the evolution of the FOM of the best individual of the population considering a general FOM defined as $BW_{\text {EO}}^2/V_\pi ^{1.8}$ for each one of the 100 initial populations. This FOM was defined based on our previous work^37^. In the present case, we did not include *IL*, since the optimization of $BW_{\text {EO}}$ already leads to lower optical losses^17^. We slightly adjusted the weight of the parameters in order to achieve a better trade-off between the efficiency and modulation speed. The FOM, however, can be modified to meet specific requirements, as will be demonstrated in “Optimization of the MZMs for different performance metrics” section.

As can be observed from Fig. 6, all the initial populations converged to a similar value of the FOM for the best individual of the population, indicating that, for this optimization problem and the aforementioned DE configuration, it would be possible to consider just one initial population. Nevertheless, we decided to use multiple initial populations to ensure that any possible influence of the initial population is avoided. Moreover, regarding the computational cost for the optimization process, the proposed methodology requires the evaluation of 5,0000,000 MZM configurations (which can be calculated by multiplying the number of initial populations, 100, the population size, 1000, and the number of iterations, 50). Thus, if each modulator configuration was simulated using the FDTD model, the optimization process would require 10,417 days (equivalent to 28 years) of continuous numerical simulations. However, employing the ANN-based model here proposed only 21 days are required to obtain the training/validation datasets, with the optimization process itself requiring only 85 seconds. Furthermore, the same dataset can be employed as a base for several optimization processes, each one aiming to design the MZM that best fits the requirements for a given application scenery.

### Optimization of the MZMs for different performance metrics

To analyze the MZM trade-off between the different metrics introduced in Sect. II.B (i.e., $BW_{\text {EO}}$, *IL*, or $V_\pi$), we impose a limit for the value of one of them, while the other two metrics were optimized using DE. In this case, the metric constraint was implemented using a penalization term, which filtered out the solutions that did not verify the limit condition. For the case of $V_\pi$ and *IL*, we define two FOMs: $\epsilon \cdot V_\pi ^{-1}$ and $\epsilon \cdot IL^{-1}$. The optimization process is executed until $V_\pi$ reaches the threshold value $V_\pi ^{thres}$ and *IL* reaches $IL^{thres}$. Once this condition is met, the FOMs are redefined as $BW_{\text {EO}}/IL$ and $BW_{\text {EO}}/V_\pi$, respectively. Similarly, for $BW_{\text {EO}}$, we define a FOM as $\epsilon \cdot BW_{\text {EO}}$, and the optimization continues until $BW_{\text {EO}}$ reaches the threshold value $BW_{\text {EO}}^{thres}$. Subsequently, the FOM becomes $1/(IL \cdot V_\pi )$. The term $\epsilon$ represents a small value, specifically $10^{-6}$, which is introduced to avoid undesired regions in the optimization space once the appropriate threshold is achieved. As a stopping condition, we adopted a maximum iteration number (in this case 50 iterations), which preliminary tests indicated was enough to ensure convergence. In addition, as we mentioned earlier, to reduce the convergence probability to suboptimal solutions, we executed the optimization algorithm with 100 independent initial populations. Following this methodology, the optimized FOMs for the MZMs are shown in Fig. 7. In each case, on which the left horizontal axis of the plots are identified in gray to indicate the constrained metric. As expected, we can note that when limiting one of the MZM metrics, there is a clear relation between the other two metrics. For example, in Fig. 7a we show the $V_\pi$ and *IL* of the MZM when the $BW_{\text {EO}}$ is limited to 30, 35, 40, and $45~\text {GHz}$. These results indicate that the larger minimum value of $BW_{\text {EO}}$ is, the larger $V_\pi$ and lower *IL* are. Moreover, the trade-off between $BW_{\text {EO}}$ and $V_\pi$ is confirmed when we limit the value of *IL*, as shown in Fig. 7b, and $V_\pi$, in Fig. 7c. This relation can be explained by noting that broader $BW_{\text {EO}}$ requires shorter MZM, which, in consequence, results in lower *IL*. In addition, since the structure is shorter, the voltage required to achieve a given phase shift is higher. Quantitatively, we can observe that a MZM with a $30~\text {GHz}$ bandwidth can be achieved with a $V_\pi$ of around $4.3~\text {V}$, but, as we increase the bandwidth up to $50~\text {GHz}$, the value of $V_\pi$ rises to almost $8~\text {V}$.

To make sure that the optimized MZM configurations were indeed outperforming the randomly generated MZMs employed to build the training and validation datasets and that the ANN did not introduce any artificial improvement, the optimized configurations in this work were simulated using the method described in “ANN-basedmodel of the MZM” section. The accuracy of the results was assessed in the same way as performed in “Analysis of the model prediction accuracy“ section for the validation set. Thus, in Fig. 5g–i, we compare the values of the metrics $BW_{\text {EO}}$, $V_\pi$, and *IL* for 50 samples, respectively, for optimized MZMs based on ANN predictions and simulations based on the electromagnetic model. Moreover, to assess the accuracy of the whole set of optimized MZMs, composed of 1,700 configurations, in Fig. 5j we show a direct comparison between the predicted via ANN and simulated $BW_{\text {EO}}$, whereas in Fig. 5k we perform the same comparison for *IL*, and in Fig. 5l we present the results for $V_\pi$. Overall, the results indicate a MSE of $0.51~\text {GHz}^2$, $0.015~\text {dB}^2$, and $0.1~\text {V}^2$ for $BW_{\text {EO}}$, $V_\pi$, and *IL*, respectively, while the Pearson correlation coefficient is above $99\%$ for all cases.

Once we ensure that the FOMs of the optimized configurations were accurately modeled by the trained ANN, we can compare the performance of optimized and randomly generated configurations. The goal is to check whether the configurations obtained using DE optimization jointly with the ANN-based MZM model present lower $V_\pi$ values for a given $BW_{\text {EO}}$ or, alternatively, for a given value of $V_\pi$, if the MZM has larger $BW_{\text {EO}}$. In the terminology of the optimization community, this is denominated as extending the Pareto front. In this sense, in order to show that the proposed method indeed shifts the Pareto front, in Fig. 7d, we show the $V_\pi$ values in terms of the $BW_{\text {EO}}$ for the complete simulated dataset, as well as the optimized configurations. In addition, we superpose the original Pareto front (identified with a red line), allowing one to notice that the points corresponding to the optimized configurations are laid close to the Pareto front. For the sake of clarity, in Fig. 7e, we present a magnified section where the points corresponding to the optimized MZM configurations can be observed in more detail, revealing that the proposed optimization relaying on ANN-modeling and DE in fact leads to improved configurations and, consequently, resulting in the shift of the Pareto front. Furthermore, the optimized MZMs represent a smoother front, which indicates that it is closer to the global boundary. In general, these results indicate that the here proposed methodology allows one to design an integrated MZM for a given set of parameters with lower values of $V_\pi$ and *IL* and/or higher values of $BW_{\text {EO}}$, improving its overall performance.

### Comparison with other optimization algorithms

Besides DE, we implemented other alternative optimization algorithms: genetic algorithm (GA)^38^, particle swarm optimization (PSO)^39^, and dual annealing (DA)^40^. The optimization process followed the approach shown in the inset of Fig 6. For the GA, we used an initial population of 10,000, selected 100 parents per iteration, set the crossover rate to 0.7, and the mutation rate to 1/8. For the PSO algorithm, we set a population size of 2,000, a moment of inertia of 0.5, and social and cognitive coefficients of 1 and 2, respectively. In the case of the DA algorithm, we configured the initial temperature to 5200 and defined the visit and acceptance parameters as 2.62 and −5, respectively. All algorithms were executed for 50 iterations.

**Figure 8:**
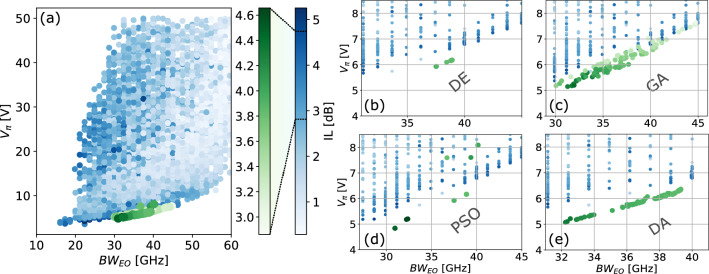
Comparison of MZMs achieved by using different optimization algorithms. (**a**) ANN training set and the MZMs obtained via DE, GA, PSO, and DA optimization algorithms. The green colorbar shows the IL range, in dB, for the optimized samples, whereas the blue colorbar represents the values for the training dataset MZMs. (**b**), (**c**), (**d**), and (**e**) shows the MZMs obtained via DE, GA, PSO, and DA, respectively, together with the surrounding training dataset samples.

Applying the same FOM as in “DE optimization employing ANN-based model” section, we obtained 100 optimized MZMs for each optimization method, corresponding to random initial conditions for each execution. Fig 8 displays the obtained results. In Fig 8a, we compare the 400 optimized MZMs with the training dataset. Fig. 8b–e depict the MZMs obtained by DE, GA, PSO, and DA, respectively. Notably, all considered algorithms yielded improved configurations compared to the training dataset. In order to quantify and compare the performance of each optimization algorithm, in Table 3, we present the average FOM for each algorithm based on 100 different executions. The DE algorithm exhibited the highest average FOM, followed by the DA algorithm. Additionally, the DE algorithm demonstrated a lower standard deviation of the FOM, indicating less variation across runs. Table 3 also displays the minimum and maximum performance parameters achieved by each algorithm. For the DE case, the $BW_{EO}$ was approximately 38 GHz, while the $V_{\pi }$ was around 6 V. Based on the more robust and consistent results obtained with DE, and in agreement with the findings in^41^, we selected DE as the preferred algorithm.

**Table 3:** Average, standard deviation, and maximum FOM obtained for each optimization algorithm considering the 100 different executions.

Algorithm	DE	GA	PSO	DA
Average FOM	56.86	51.06	48.4	56.5
FOM standard deviation	0.17	2.31	9.8	0.53
Maximum FOM	57.09	54.86	57.15	57.11
$BW_{EO}$ [GHz] min/max	37.5/38.9	30/44.8	30.9/40.1	32.2/39.3
IL [dB] min/max	3.8/3.9	2.9/4.4	3.8/4.65	3.8/4.3
$V_{\pi }$ [V] min/max	5.9/6.2	5.1/7.6	4.8/8.1	5.1/6.4

The table also shows the minimum and maximum obtained $BW_{EO}$, IL, and $V_{\pi }$ for each algorithm.

## Conclusions

Due to its compatibility with CMOS, Si photonics has emerged as a high-potential platform for the implementation of MZMs. However, because of the weak electro-optic effects of Si, MZMs employing this technology must be carefully optimized exploring the largest possible number of design parameters to achieve the best overall performance. To achieve this goal, in this paper, we proposed an optimization approach based on ANNs and DE. For that, in the first stage, we used a consolidated simulation model to acquire a dataset, which was then used to train and evaluate different ANN-based models to predict the value of *IL*, $BW_{\text {EO}}$, and $V\pi$ in terms of 8 constitutive and operational parameters. Among the considered configurations, we found that a 5-layer MLP with DO, BN, and RCs is the best approach to reduce the MSE of the outputs for this specific problem. Moreover, the obtained results indicate that the developed model showed high prediction accuracy requiring an inference time 7 orders of magnitude lower than the traditional simulation in a general-purpose workstation. Such drastic reduction of the execution time, enabled the application of multi-agent optimization, in particular DE, with large population sizes, as well as tuning the optimization parameters. The results achieved using the proposed combination of ANN modeling and DE optimization allowed the generation of novel MZM configurations, which outperform randomly generated combinations of its design parameters. This is, to our best knowledge, the first time that ANNs are employed to design integrated MZMs. Although the obtained results are interesting and show the feasibility and potential of the proposed design method, the work could be extended to more sophisticated MZM models, for example including the electrode-related parameters, or to test other heuristic optimization algorithms, such as particle swarm optimization or genetic algorithms. Future works will present a system performance analysis of the optimized modulator, including experimental results.

## Data Availability

The datasets generated and/or analysed during the current study are available in the Figshare repository, https://figshare.com/s/2f4c54659bb3e18f19a3.
